# Inhibition of NLRP3 inflammasome by thioredoxin-interacting protein in mouse Kupffer cells as a regulatory mechanism for non-alcoholic fatty liver disease development

**DOI:** 10.18632/oncotarget.17489

**Published:** 2017-04-27

**Authors:** Kun He, Xiwen Zhu, Yan Liu, Chunmu Miao, Tao Wang, Peizhi Li, Lei Zhao, Yaxi Chen, Junhua Gong, Can Cai, Jinzheng Li, Shengwei Li, Xiong Z. Ruan, Jianping Gong

**Affiliations:** ^1^ Department of Hepatobiliary Surgery, The Second Affiliated Hospital of Chongqing Medical University, Chongqing, China; ^2^ Centre for Lipid Research, Key Laboratory of Molecular Biology on Infectious Diseases, Ministry of Education, The Second Affiliated Hospital of Chongqing Medical University, Chongqing, China; ^3^ Centre for Nephrology, University College London (UCL) Medical School, Royal Free Campus, London, United Kingdom; ^4^ Department of Gastroenterology and Hepatology, The Second Affiliated Hospital of Chongqing Medical University, Chongqing, China

**Keywords:** NLRP3 inflammasome, Kupffer cell, non-alcoholic fatty liver disease, IL-1β

## Abstract

NOD-like receptor (NLR) NLRP3 inflammasome activation has been implicated in the progression of non-alcoholic fatty liver disease (NAFLD) from non-alcoholic fatty liver (NAFL) to non-alcoholic steatohepatitis (NASH). It has been also shown that palmitic acid (PA) activates NLRP3 inflammasome and promotes interleukin-1β (IL-1β) secretion in Kupffer cells (KCs). However, the specific mechanism of the NLRP3 inflammasome activation is unclear. We studies the molecular mechanisms by investigating the roles of Thioredoxin-interacting protein (TXNIP) and NLRP3 on NAFLD development in patients, high-fat diet (HFD)-induced NAFL and methionine choline deficient (MCD) diet-induced NASH in wild type (WT), TXNIP^−/−^ (thioredoxin-interacting protein) and NLRP3^−/−^ mice, and isolated KCs. We found that the expressions of NLRP3 and TXNIP in human liver tissues were higher in NASH group than in NAFL group. Furthermore, co-immunoprecipitation analyses show that activation of the TXNIP-NLRP3 inflammasome protein complex occurred in KCs of NASH WT mice rather than NAFL WT mice, thus suggesting that the formation and activation of this protein complex is mainly involved in the development of NASH. NLRP3^−/−^ mice exhibited less severe NASH than WT mice in MCD diet model, whereas TXNIP deficiency enhanced NLRP3 inflammasome activation and exacerbated liver injury. PA triggered the activation and co-localization of the NLRP3 inflammasome protein complex in KCs isolated from WT and TXNIP^−/−^ but not NLRP3^−/−^ mice, and most of the complex co-localized with mitochondria of KCs following PA stimulation. Taken together, our novel findings indicate that TXNIP plays a protective and anti-inflammatory role in the development of NAFLD through binding and suppressing NLRP3.

## INTRODUCTION

Non-alcoholic fatty liver disease (NAFLD) is considered to be the main hepatic manifestation of metabolic syndrome, and is currently the most common cause of chronic liver disease in many developed and developing countries worldwide [1, 2]. NAFLD ranges from hepatic steatosis or non-alcoholic fatty liver (NAFL), which is characterized by the accumulation of triglycerides in hepatocytes, to non-alcoholic steatohepatitis (NASH), which can eventually progress to end-stage liver diseases such as cirrhosis and hepatocellular carcinoma [1, 3]. The pathogenesis of NAFLD is commonly based on the ‘double-hit’ hypothesis, which was proposed by Day and James in 1998 [4]. According to this theory, the initial hepatocellular lipid accumulation represents the ‘first hit’, followed by a “second hit”, in which pro-inflammatory mediators induce inflammation and hepatocellular injury, thus promoting the progression to NASH. Recently, increasing emphasis has been placed on the mechanisms underlying the progression from NAFL to NASH, which has been deemed the crucial step in the development of NAFLD [5]. It is currently thought that the genesis and development of NASH are closely related to metaflammation, a low-grade form of chronic inflammation that is mainly involved in the “second hit” [6]. However, the exact mechanism underlying the development of NASH remains to be clearly defined.

NLRP3 is the best characterized member of the inflammasome family. It consists of the NOD-like receptor (NLR) NLRP3, the adaptor molecule apoptosis-associated speck-like protein containing a caspase recruitment domain (ASC) and the effector molecule pro-caspase-1 [7]. NLRP3 inflammasome activation is a two-step process [8]. The first step consists of cell priming with an NF-κB activator, leading to the up-regulation of NLRP3 and pro-IL-1β expression, and the second step includes a broad variety of activators, such as free fatty acid (FFA), which induces NLRP3 inflammasome activation [8]. The key step in NLRP3 inflammasome activation is the cleavage and activation of Caspase-1, which subsequently activates pro-IL-1β to IL-1β, and has been shown to play an essential role in the formation of NASH [9, 10].

Importantly, high plasma levels of saturated FFAs, such as palmitic acid (PA), which has been suggested to trigger inflammation and contribute to the development of NASH, have been reported in mice models of methionine choline deficient (MCD) diet-induced NASH and NASH patients [11]. Kupffer cells (KCs) are the liver-derived macrophages that represent approximately 80–90% of the tissue resident macrophages [12, 13]. KCs have been implicated in the pathogenesis of various liver diseases, such as NASH, alcoholic liver disease and liver fibrosis, and are thought to contribute to liver rejection after transplantation [14–16]. KCs have been described as the primary site of NLRP3 inflammasome activation and the source of the pro-inflammatory cytokine IL-1β in liver tissues [17]. Thioredoxin-interacting protein (TXNIP, also known as VDUP1), which has been implicated in many metabolic diseases, is an essential intermediate that is associated with NLRP3 activation through binding to NLRP3 in a reactive oxygen species (ROS)-dependent manner after its detachment from thioredoxin (TRX) [18]. In addition, mitochondria are the main cellular source of ROS, which have been proven to form a central component proximal to NLRP3 inflammasome activation [19, 20]. However, the roles of the TXNIP and NLRP3 inflammasome in the progression of NAFLD are controversial and the precise mechanism underlying NLRP3 inflammasome activation through TXNIP in KCs remains to be evaluated [21].

In this study, we explored the roles and relationship of TXNIP and NLRP3 in NLRP3 inflammasome activation in NAFLD patients, high-fat diet (HFD)-induced NAFL and MCD diet-induced NASH in wild type (WT), TXNIP knockout (TXNIP^−/−^) and NLRP3^−/−^ mice, and in isolated KCs.

## RESULTS

### NASH patients presented increased expressions of NLRP3 and TXNIP and increased pro-inflammatory cytokine IL-1β levels

We examined the serological and liver pathological changes in patients with NAFL and NASH. The patient characteristics are summarized in Table 1. There were no significant differences in alanine aminotransferase (ALT), aspartate transaminase (AST) and triglyceride (TG) levels among the groups, whereas the serum levels of FFA and IL-1β were significantly higher in the NASH patients compared with the normal healthy controls and NAFL patients, thus suggesting that the FFA and IL-1β levels are mainly associated with NASH but not NAFL.

**Table 1:** Comparison of the clinical and demographic factors

Characteristic	Normal	NAFL	NASH
Case number	5	6	6
Age (years)	50 ± 7	48.2 ± 6.5	46 ± 6
Gender (male/female)	3/2	3/3	3/3
ALT (U/L)	15.33 ± 3.06	28.80 ±12.70	25.75 ±13.45
AST (U/L)	16.00 ± 1.73	24.40 ± 4.56	24.75 ± 12.45
TG (mmol/L)	0.76 ± 0.27	1.70 ± 1.21	1.90 ± 0.73
FFA (mmol/L)	0.63 ± 0.21	0.79 ± 0.11	2.11 ± 0.44*
IL-1β (ng/l)	Non-detectable	Non-detectable	27.23 ± 6.71*
NAS	0	2 ± 0.71	5 ± 0.82

Abbreviations: ALT, alanine aminotransferase; AST, aspartate aminotransferase; TG, triglycerides; FFA, free fatty acid; NAS, NASH activity score. *P < 0.05.

**Figure 1:**
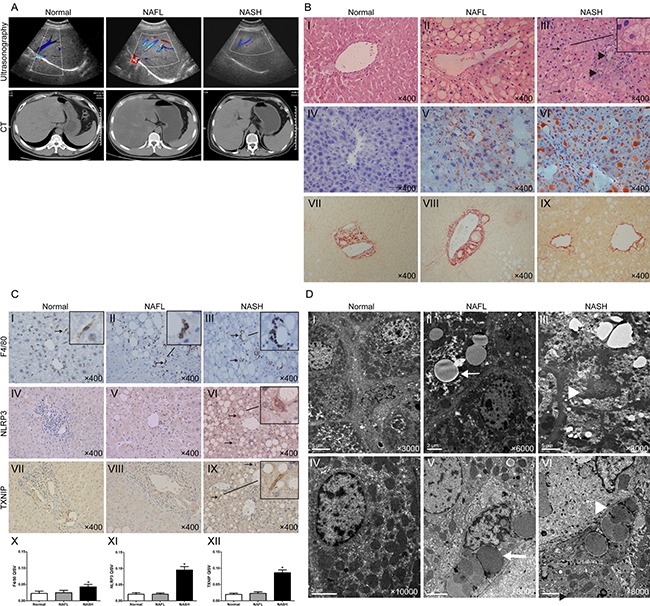
Human NASH is characterised by increased expressions of F4/80, NLRP3 and TXNIP (**A**) Abdominal ultrasonography and CT scan images of normal, NAFL, and NASH patients. (**B**) HE, Oil Red O, and Sirius Red staining of liver sections (arrows, ballooning hepatocytes; arrowheads, inflammatory cells). (**C**) Immunohistochemical staining for F4/80, NLRP3 and TXNIP in liver sections (arrows, positive KCs). The quantitative immunohistochemical staining values (QISVs) were analysed for F4/80, NLRP3, and TXNIP protein expression (*n* = 5 for normal, *n* = 6 for NAFL, and *n* = 6 for NASH). *P < 0.05 for NASH vs NAFL and normal groups. (**D**) TEM images of human livers (arrows, lipid droplets; arrowhead in Panel III, hepatocyte oedema, in Panel VI, fat-storing cell). The original magnification is labeled in each picture.

In the NASH patients, liver ultrasonography showed increased echogenicity and unequal distribution, and computer tomographic images revealed normal sized but hypoechogenic livers, which is a typical imaging manifestation of NAFLD (Figure 1A). Hematoxylin-eosin (HE) and Oil Red O staining showed that the NAFL livers exhibited obvious steatosis, as compared with the normal livers (Figure 1B II vs I). The NASH livers had grades of steatosis similar to livers with NAFL (Figure 1B III vs II), whereas hepatocyte ballooning (Figure 1B III, arrows) and inflammatory cell infiltration (Figure 1B III, arrowheads) were more severe. Sirius Red staining showed that none of the groups exhibited liver fibrosis (Figure 1B, VII-IX). Immunohistochemical staining showed that the numbers of F4/80-, NLRP3- and TXNIP-positive cells were similar between the normal and NAFL groups (Figure 1C II vs I, V vs IV, VIII vs VII), whereas more F4/80-, NLRP3- and TXNIP-positive cells were observed in the NASH group as indicated by arrows than in the NAFL group (Figure 1C III, VI, IX, X-XII). The ultrastructures of the liver samples were examined through transmission electron microscopy (TEM), which revealed a significant increase in lipid droplets (arrows) in the hepatocyte cytoplasm accompanied by a compressed hepatic sinusoid in the NAFL group (Figure 1D II and V). Extreme hepatocyte oedema (arrowhead) filled with lipid droplets (arrow) was observed in the NASH group (Figure 1D III). A fat-storing cell was found in a NASH liver sample (Figure 1D VI, arrowhead). Taken together, these results for human samples demonstrated that serum FFA might act as a type of danger-associated molecular pattern (DAMP) that activates TXNIP and the NLRP3 inflammasome in KCs and subsequently stimulates KCs to secrete IL-1β and promotes the progression from NAFL to NASH.

### NLRP3 and TXNIP are involved in the development of NAFLD in mice

**Figure 2:**
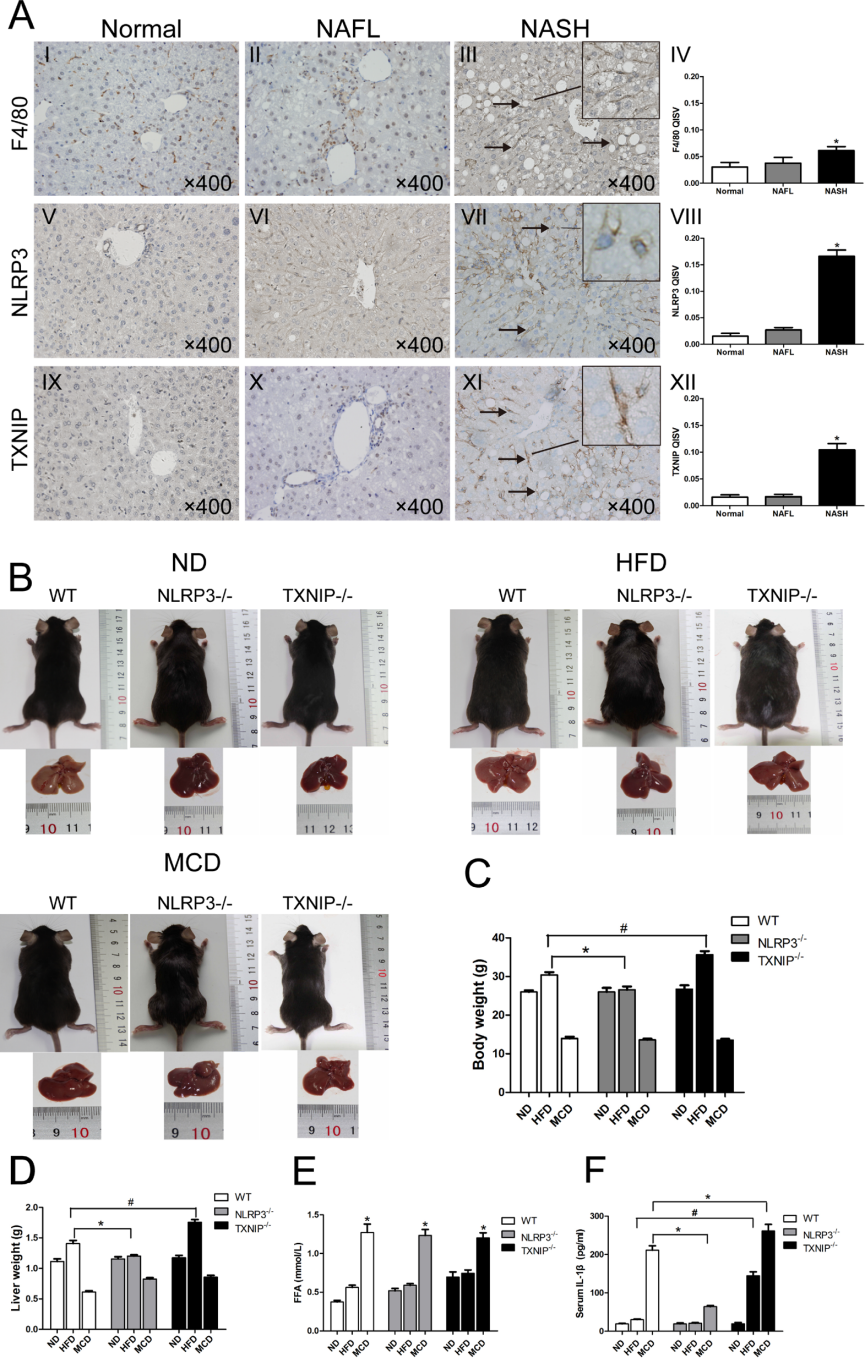
KCs infiltration and expressions of NLRP3 and TXNIP are elevated in NASH livers of WT mice (**A**) Immunohistochemical staining for F4/80, NLRP3 and TXNIP in liver sections (arrows, positive KCs). The quantitative immunohistochemical staining values (QISVs) were analysed for F4/80, NLRP3, and TXNIP protein expression. (**B**) Images of mice and liver phenotypes from WT, NLRP3^−/−^ and TXNIP^−/−^ mice fed ND, HFD or MCD diets. (**C**, **D**) Body and liver weights after intervention. (**E**, **F**) Serum FFA and IL-1β concentrations. Data are expressed as the mean ± SD. *P < 0.05, ^#^*P* < 0.05. The original magnification and scale bars are labeled in each picture.

We used diet-induced models of NAFL and NASH to detect the roles of NLRP3 and TXNIP in NAFL and NASH development. Immunohistochemical staining revealed a greater number of F4/80-, NLRP3- and TXNIP-positive cells in the livers of WT mice in NASH group (arrows) than in normal and NAFL groups, whereas there were no significant differences in these cell populations between NAFL and normal groups (Figure 2A). Next, we used both NLRP3^−/−^ and TXNIP^−/−^ mice to determine the roles of NLRP3 and TXNIP during NAFLD development by using HFD-induced NAFL and MCD diet-induced NASH models. The body weights of the mice in our diet-induced models were weekly determined (Supplementary Figure 1). There were no significant difference in body weights among the ND-fed WT, NLRP3^−/−^ and TXNIP^−/−^ mice (Figure 2B and 2C). NLRP3^−/−^ mice gained less body weight than the WT mice after HFD feeding, while TXNIP^−/−^ mice gained more body weight than the WT mice (Figure 2B and 2C). All animals fed MCD had decreased body weights (Figure 2B and 2C). Liver weights of WT mice in the HFD group were heavier than the livers from NLRP3^−/−^ mice, whereas Liver weights of TXNIP^−/−^ mice in the HFD group were heavier than the livers from WT mice. The liver weights in the MCD groups also decreased after intervention (Figure 2B and 2D). It is noticeable that TXNIP^−/−^ mice gained more body and liver weight than the WT mice after HFD feeding (Figure 2B, 2C and 2D), thus indicating that TXNIP deficiency may aggravate NAFL severity. The FFA levels were all significantly elevated in these MCD groups, and there is no significant difference among these MCD groups (Figure 2E). In these MCD groups, NLRP3^−/−^ mice showed the lowest IL-1β levels (Figure 2F). Nevertheless, the IL-1β levels in HFD and MCD groups of TXNIP^−/−^ mice were significantly higher than those in the corresponding groups of WT mice, which also indicated that knocking out TXNIP can exacerbate liver injury, and this injury started from normal liver to NAFL (Figure 2F).

### NLRP3 and TXNIP participate in different stages of NAFLD progression

**Figure 3:**
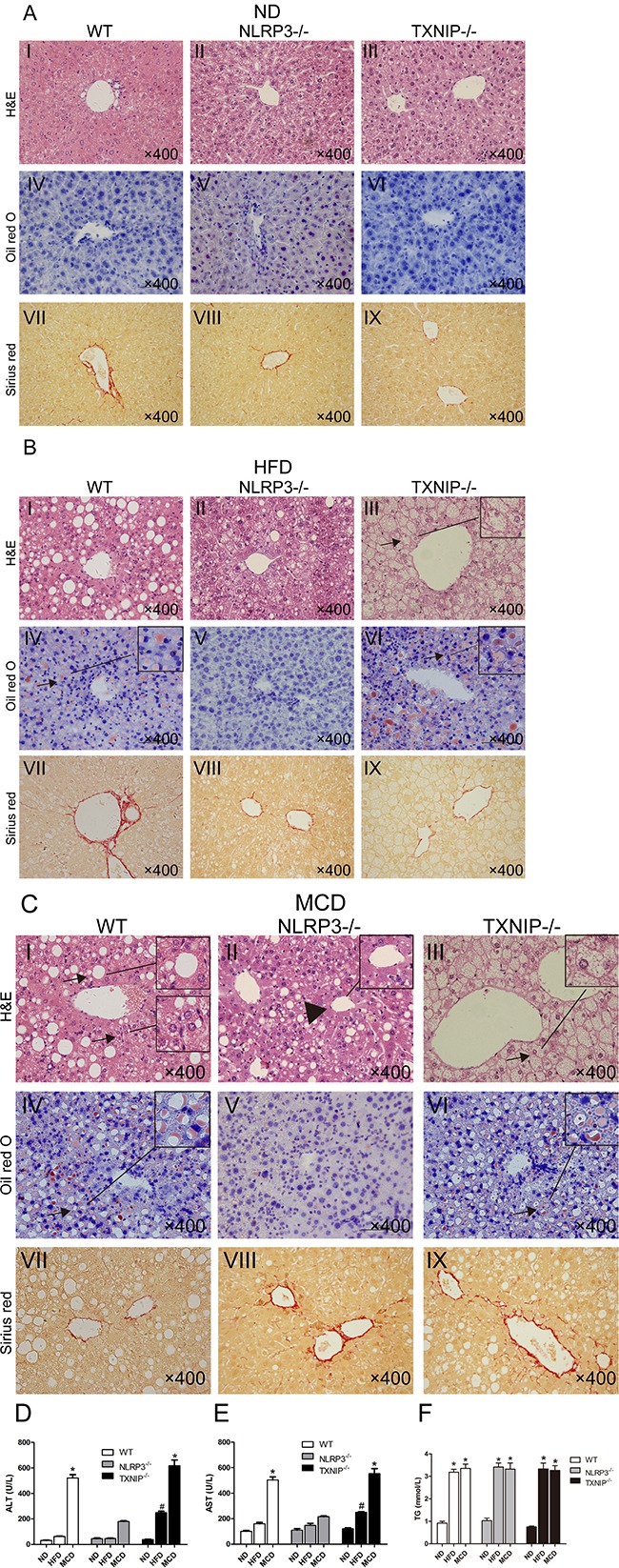
NLRP3^−/−^ mice exhibit reduced hepatocyte injury in MCD group and TXNIP knockout results in exacerbated hepatocyte injury after HFD feeding (**A**, **B**, **C**) Images of HE, Oil Red O and Sirius Red staining of liver sections from WT, NLRP3^−/−^ and TXNIP^−/−^ mice fed ND, HFD or MCD diets. (**D**, **E**) Serum ALT and AST levels. (**F**) Serum TG levels. Data are expressed as the mean ± SD. *represents *P* < 0.05 when comparing MCD groups of WT and TXNIP^−/−^ genotypes with NLRP3^−/−^ mice, ^#^represents *P* < 0.05 when comparing HFD groups of TXNIP^−/−^ genotypes with WT mice. The original magnification and scale bars are labeled in each picture.

No significant differences were observed in liver pathological characteristics among the three types of mice fed ND (Figure 3A, I, II, and III). Although HFD groups of WT and NLRP3^−/−^ mice showed similar grades of liver steatosis (Figure 3B, I and II), hepatocyte ballooning and inflammatory cell infiltration were markedly attenuated in the MCD-fed group of NLRP3^−/−^ mice (Figure 3C, II vs I, arrows, hepatocyte ballooning and inflammatory cells, arrowhead, normal area). Furthermore, we found that TXNIP^−/−^ mice had a susceptibility to HFD, and the TXNIP^−/−^ mice can be induced to NASH by HFD feeding alone, which is much more severe than HFD groups of WT mice and NLRP3^−/−^ mice (Figure 3B, III vs I and II, arrow, hepatocyte ballooning). Interestingly, the main pathological characteristic of TXNIP^−/−^ mice fed with HFD and MCD was hepatocyte ballooning (Figure 3B, III, Figure 3C, III), which is also a typical pathological characteristic of NASH in addition to inflammatory cell infiltration. This finding suggested that TXNIP might be mainly involved in the suppression of hepatocyte ballooning formation during the development of NASH. Sirius Red staining indicated that none of the groups displayed significant liver fibrosis (Figure 3A, 3B and 3C). In these MCD groups, NLRP3^−/−^ mice showed the lowest values of plasma biomarkers of liver damage (ALT and AST). Nevertheless, the levels of ALT and AST in HFD group of TXNIP^−/−^ mice were significantly higher than in the corresponding groups of WT mice, thus suggesting that TXNIP deficiency can exacerbate liver injury induced by HFD (Figure 3D and 3E). As expected, HFD and MCD feeding increased serum TG levels of WT mice, and there was no significant difference between the two groups. It is interesting that NLRP3 deficiency and TXNIP deficiency have no effect on the serum TG levels induced by HFD and MCD feeding (Figure 3F). Taken together, these results suggest that NLRP3 plays a pro-inflammatory role mainly in the progression from NAFL to NASH and that TXNIP has anti-inflammatory and protective effects from the stage of NAFL formation.

The ultrastructures of livers from WT mice were observed through TEM. In the ND group, the morphology and ultrastructures of the hepatocytes and the hepatic sinusoid were normal. Lipid droplets were apparent in hepatocytes, and the hepatic sinusoid was compressed in HFD group. In addition, the integrity of the hepatocytes and the hepatic sinusoid were destroyed in MCD livers (Supplementary Figure 2).

### TXNIP and NLRP3 inflammasome participate in the progression of NASH through forming a protein complex

**Figure 4:**
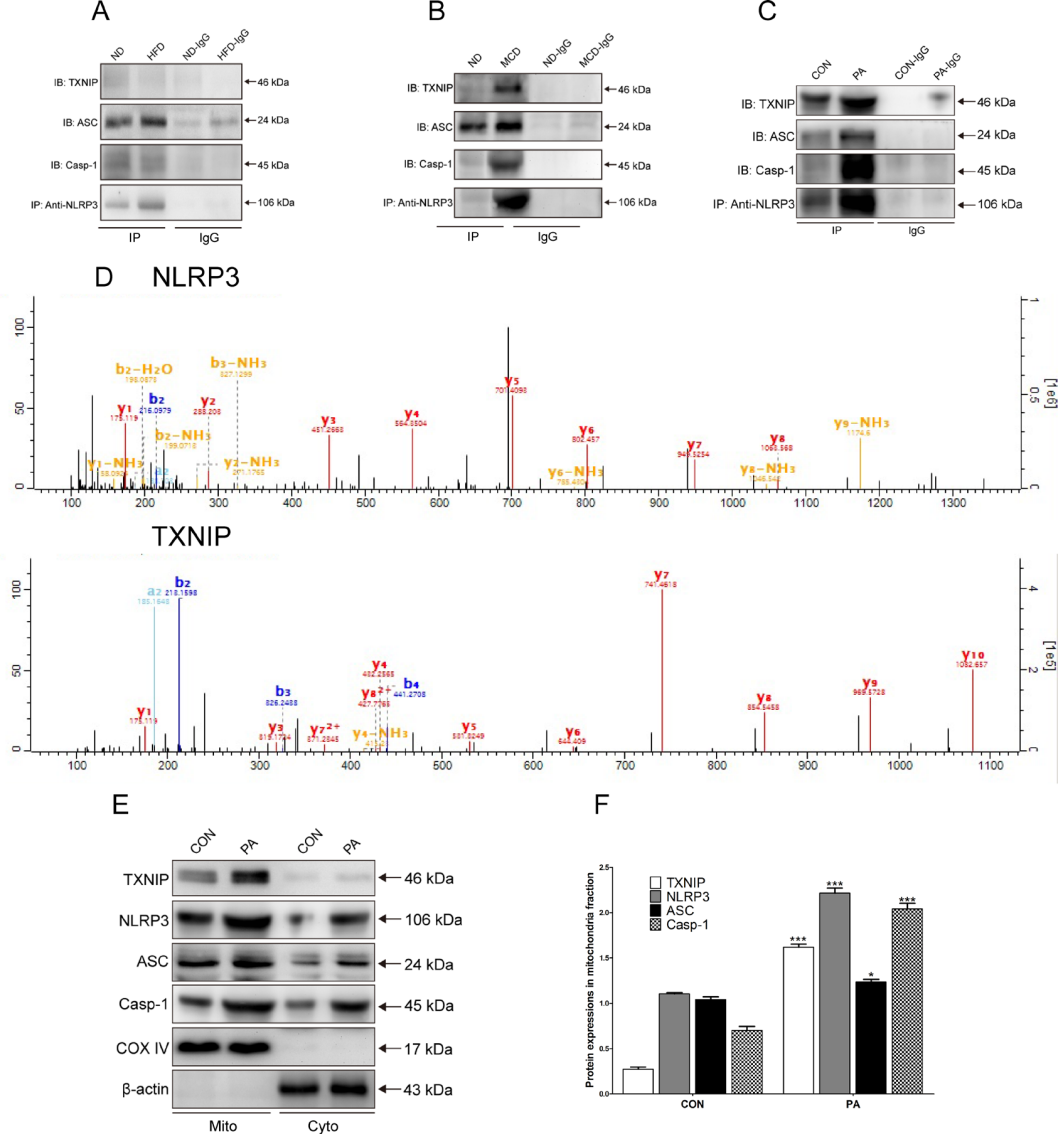
TXNIP-NLRP3 inflammasome complex is formed and co-localizes in the mitochondria *in vivo* and *in vitro* in WT mice (**A**) Protein expressions levels as determined by Co-IP of TXNIP, NLRP3, ASC and Caspase-1 proteins from WT mice KCs of normal and NAFL groups. (**B**) Protein expressions levels as determined by Co-IP of TXNIP, NLRP3, ASC and Caspase-1 proteins from WT mice KCs of normal and NASH groups. (**C**) Co-IP of NLRP3 with TXNIP, ASC and Caspase-1 assessed by western blotting in KCs lysates from WT mice in the CON and PA groups. (**D**) Mass-spectrograms of NLRP3 and TXNIP. (**E**, **F**) Protein expression levels of TXNIP and NLRP3 inflammasome proteins in mitochondrial and cytosolic fractions. The histogram represents the mean ± SD of the densitometric scans for target protein bands normalized by comparison to COX4 or β-actin. *represents *P* < 0.05 when comparing PA with CON group. ***represents *P* < 0.001 when comparing PA with CON group.

To investigate whether NLRP3 inflammasome complex is mainly activated in NASH, we performed Co-IP experiments. The results showed a very low level of protein complex formation in the KCs from WT mice in the normal and NAFL groups (Figure 4A), whereas a large amount of the complex was presented in the NASH group, and TXNIP is part of the complex (Figure 4B). These results indicated that TXNIP-NLRP3 inflammasome protein complex in KCs from WT mice contributed to the progression of NASH but not NAFL. We also investigated the potential relationship between TXNIP and NLRP3 *in vitro*. We used the KCs from WT mice stimulated with PA to investigate the interaction between TXNIP and NLRP3 by Co-IP. The results showed that the amount of TXNIP-NLRP3 inflammasome protein complex was much greater in the PA group than in the control (CON) group (Figure 4C). Furthermore, the high performance liquid chromatography-mass spectrometry (LC-MS) results confirmed that TXNIP and NLRP3 were both detected in the IP groups and there were differences among the IP groups (Figure 4D).

### TXNIP and NLRP3 inflammasome complex co-localize with the mitochondria in KCs

To determine the essential role of mitochondria in the process of NLRP3 inflammasome activation, we evaluated the expressions of TXNIP and NLRP3 inflammasome in the mitochondrial and cytosolic fractions of KCs by western blotting. The results showed that the expression levels of TXNIP and NLRP3 inflammasome were significantly elevated in the mitochondrial fraction of each group compared with the cytosolic fraction, thus suggesting that TXNIP and NLRP3 inflammasome mainly co-localized with the mitochondria after PA stimulation (Figure 4E, 4F). In the mitochondrial fraction, PA stimulation increased the expression of NLRP3 inflammasome compared with that in CON group. The expression of NLRP3 was minimal in resting KCs, and was clearly elevated in response to PA. The expression levels of ASC and Caspase-1 in the PA group were also higher than those in the CON group (Figure 4E, 4F). These results demonstrated that the TXNIP-NLRP3 inflammasome complex mainly co-localized with mitochondria after PA stimulation.

### TXNIP plays a protective role in the progression of NAFLD *in vivo*

**Figure 5:**
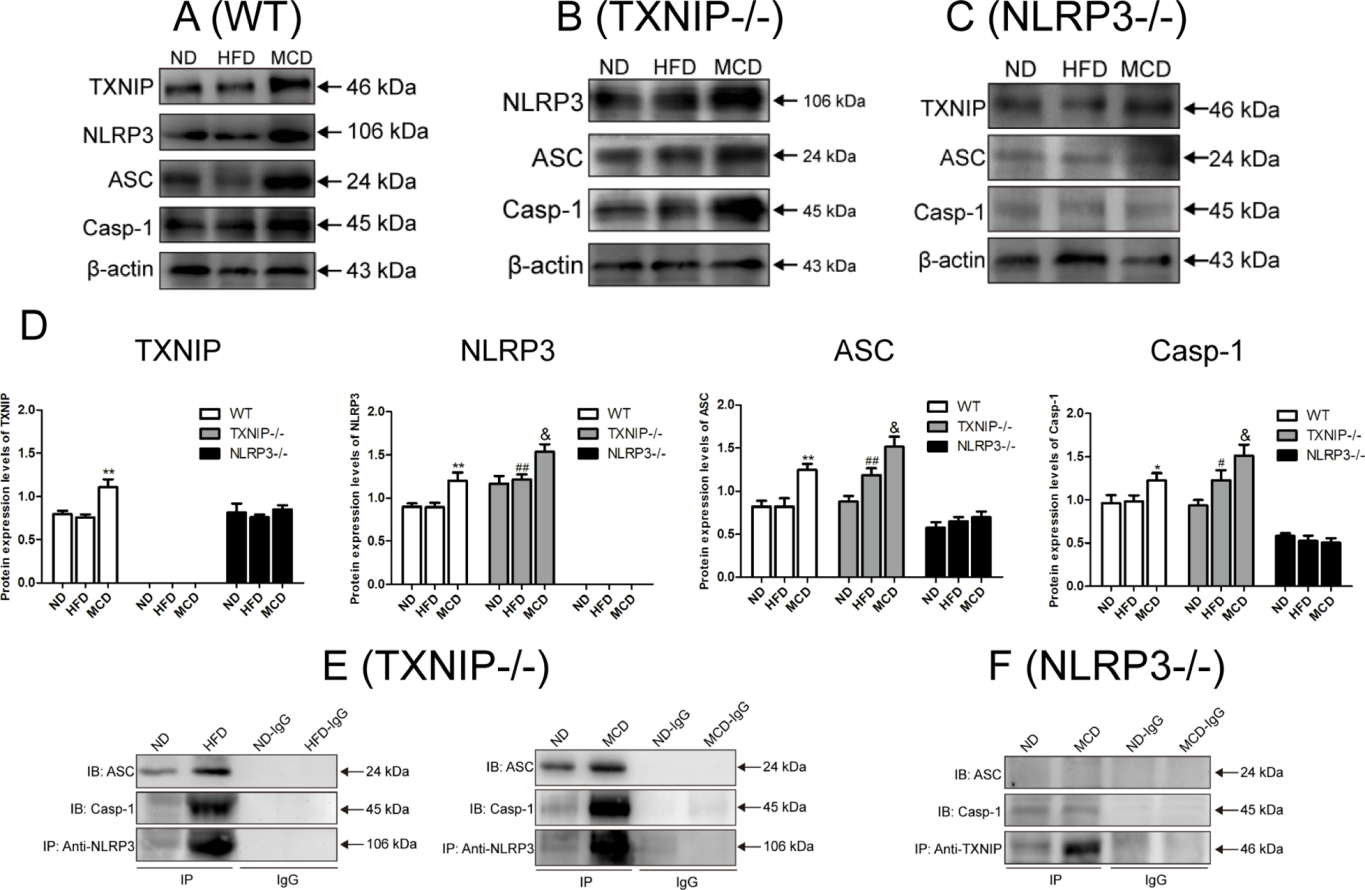
NLRP3 inflammasome activation in KCs promotes NAFL to NASH progression Analysis of isolated KCs from WT, TXNIP^−/−^ and NLRP3^−/−^ mice fed ND, HFD or MCD diets. TXNIP and NLRP3 inflammasome protein expressions in the KCs from (**A**) WT mice, (**B**) TXNIP^−/−^ mice, and (**C**) NLRP3^−/−^ mice. (**D**) Statistical analysis of protein expressions for TXNIP, NLRP3, ASC, and Caspase-1 in KCs from WT, TXNIP^−/−^ and NLRP3^−/−^ mice. (**E**, **F**) Protein concentration by Co-IP of TXNIP, NLRP3, ASC and Caspase-1 proteins in KCs from TXNIP^−/−^ and NLRP3^−/−^ mice. The histogram represents the mean ± SD of the densitometric scans for target protein bands normalized by comparison to β-actin. *represents *P* < 0.05 when comparing MCD with ND and HFD groups. **represents *P* < 0.01 when compare MCD with ND and HFD groups. ^#^represents *P* < 0.05 when comparing HFD group of TXNIP^−/−^ mice with WT mice. ^##^ represents *P* < 0.01 when comparing HFD groups of TXNIP^−/−^ mice with WT mice. ^&^ represents *P* < 0.05 vs corresponding groups of WT mice.

As demonstrated in Figures 1 and 2, KCs play an important role in liver innate immunity. We next studied TXNIP and NLRP3 inflammasome protein expressions in KCs from livers of normal, NAFL and NASH mice to investigate the potential relationship between TXNIP and NLRP3. Our results showed that the expressions of TXNIP, NLRP3, ASC and Caspase-1 were all elevated in KCs from WT mice fed MCD diet, as compared with the levels in ND and HFD groups, whereas no significant differences were observed between ND and HFD groups (Figure 5A, 5D). These results suggested that TXNIP, NLRP3, ASC and Caspase-1 mainly participate in the progression from NAFL to NASH but not in the initiation of NAFL in WT mice and are consistent with the results described in Figures 1, 2, 3, 4. Moreover, it is noticeable that TXNIP deficiency significantly increased the expressions of NLRP3, ASC and Caspase-1 in KCs from both HFD and MCD groups compared with corresponding groups of WT mice (Figure 5B, 5D). There were no increases in TXNIP or NLRP3 inflammasome expression in KCs from MCD-fed NLRP3^−/−^ mice (Figure 5C, 5D).

We further examined the effects of TXNIP and NLRP3 deficiency on NLRP3 inflammasome formation and activation in KCs from knockout mice by using Co-IP. Unexpectedly, NLRP3 inflammasome was formed and activated in KCs from TXNIP^−/−^ mice in the HFD group, and the activation of NLRP3 inflammasome was more apparent in KCs from TXNIP^−/−^ mice in the MCD group (Figure 5E) than in KCs from WT mice (Figure 4B), whereas NLRP3 protein complex expression decreased significantly in the KCs from NLRP3^−/−^ mice (Figure 5F). Taken together, these results demonstrated that NLRP3 was essential for NLRP3 inflammasome activation in KCs in a MCD-diet induced NASH model, and that TXNIP suppressed NLRP3 inflammasome formation and activation in KCs from WT mice fed by HFD and MCD diet, as evidenced by the result that TXNIP knockout significantly up-regulated the expression and activation NLRP3 inflammasome in KCs, which could explain the results in Figure 3B that TXNIP knockout can lead to NASH from HFD alone.

### TXNIP inhibits NLRP3 inflammasome activation and IL-1β secretion *in vitro*

**Figure 6:**
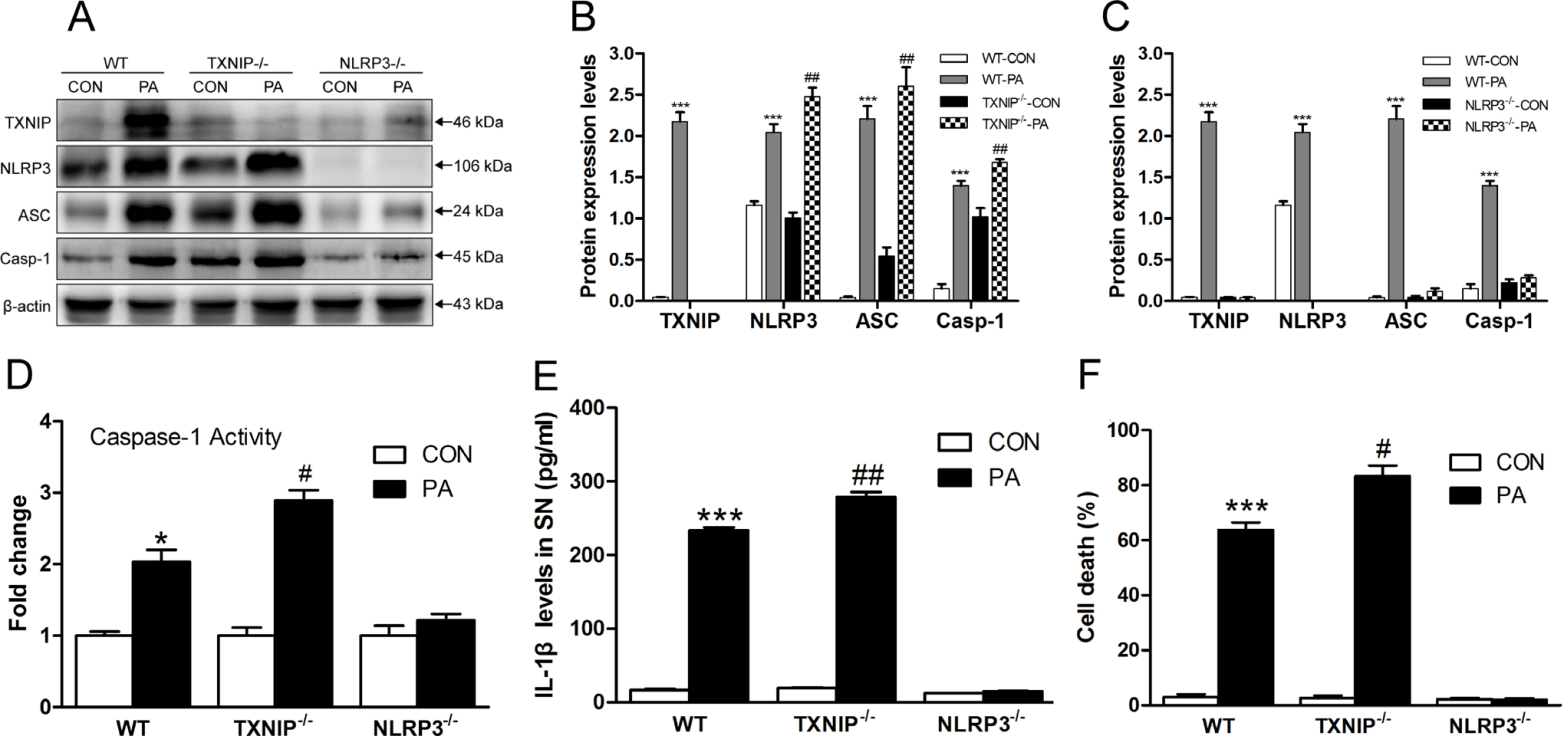
TXNIP inhibits NLRP3 inflammasome activation and IL-1β secretion *in vitro* (**A**) TXNIP and NLRP3 inflammasome protein expressions in KCs of (**B**) WT and TXNIP^−/−^ and (**C**) WT and NLRP3^−/−^ mice. (**D**) Caspase-1 activity in KCs. (**E**) IL-1β levels in the supernatant (SN) from KCs of WT, TXNIP^−/−^ and NLRP3^−/−^ mice. (**F**) NLRP3 deficiency blocks pyroptosis. The histogram represents the mean ± SD of the densitometric scans for target protein bands normalized by comparison to β-actin. ***represents *P* < 0.001, when comparing PA group with CON for each genotypic group. ^#^represents *P* < 0.05 when comparing PA groups of TXNIP^−/−^ group with the corresponding WT group. ^##^represents *P* < 0.01 when comparing PA groups of TXNIP^−/−^ group with the corresponding WT group.

We isolated and cultured KCs from WT, NLRP3^−/−^ and TXNIP^−/−^ mice in the absence or presence of PA, an established NLRP3 activator, to evaluate the protective role of TXNIP on the progression of NAFLD *in vitro* by analyzing the expressions of NLRP3 inflammasome components and IL-1β secretion in KCs. We found that PA increased TXNIP, NLRP3, ASC and Caspase-1 protein levels in the KCs of WT mice (Figure 6A and 6B). Interestingly, PA induced a greater up-regulation of NLRP3, ASC and Caspase-1 protein levels in the KCs from TXNIP^−/−^ mice than in those from WT mice (Figure 6A and 6B), thus supporting the result from *in vivo* experiments that TXNIP provides a protective role by suppressing NLRP3 protein expression induced by FFA. As expected, PA had no effect on ASC and Caspase-1 protein expression in the KCs from NLRP3^−/−^ mice (Figure 6A and 6C).

We also found that caspase-1 activity was significantly increased in the KCs from WT and TXNIP^−/−^ mice stimulated by PA, and the level in PA group of TXNIP^−/−^ group was higher than WT group (Figure 6D). In agreement with the increased inflammasome expression and caspase-1 activity, IL-1β secretion was significantly increased in the supernatant (SN) of KCs from WT mice subjected to PA treatment. As a consequence of TXNIP knockout, the level of IL-1β in PA group was higher than PA group of WT mice. There were no significant differences between CON and PA groups in the SN of KCs from NLRP3^−/−^ mice (Figure 6E). In order to investigate the consequence in terms of pyroptosis, we determined the percentage of cell death in KCs from WT, TXNIP^−/−^, and NLRP3^−/−^ mice with or without PA stimulation. As expected, PA could trigger pyroptosis in KCs from WT mice. TXNIP deficiency exacerbates pyroptosis in KCs under PA stimulation, whereas there is no significant difference between the control and PA groups in KCs from NLRP3^−/−^ mice (Figure 6F).

**Figure 7:**
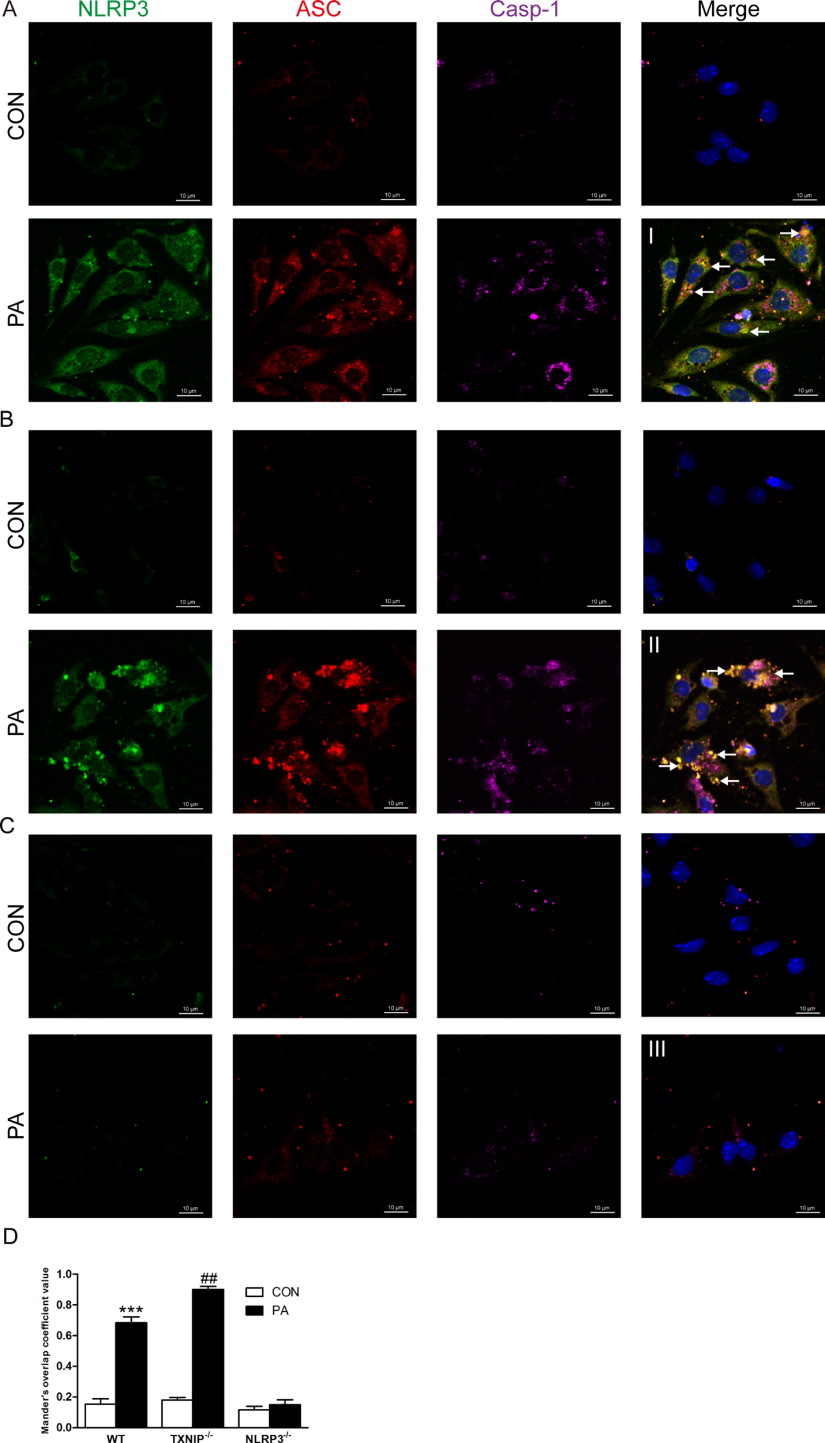
TXNIP deficiency enhances the co-localization of NLRP3, ASC and Caspase-1 in KCs NLRP3 inflammasome formation in KCs as shown by confocal microscopy from (**A**) WT, (**B**) TXNIP^−/−^, and (**C**) NLRP3^−/−^ mice (arrows, co-localization of NLRP3, ASC and Caspase-1). (**D**) The Mander's overlap coefficient values were analysed for the degree of co-localization. ***represents *P* < 0.001, when comparing PA group with CON group. ^##^represents *P* < 0.01 when comparing PA groups of TXNIP^−/−^ group with the corresponding WT group. Scale bars are labeled in each picture.

Finally, the immunofluorescence results showed that PA stimulation increased the expressions and co-localization of NLRP3 inflammasome in KCs from WT mice (Figure 7A, 7D, 7I, arrows, co-localization of NLRP3, ASC and Caspase-1). More importantly, the results revealed a much more co-localization in PA-stimulated KCs from TXNIP^−/−^ mice compared with those from WT mice (Figure 7B, 7D, II, arrows, co-localization of NLRP3, ASC and Caspase-1). As expected, PA stimulation had no obvious effect on KCs from NLRP3^−/−^ mice (Figure 7C, 7D, III). Taken together, these results demonstrated that PA mechanistically acts as a DAMP and increases the expressions of TXNIP and NLRP3 inflammasome as well as IL-1β secretion in KCs. Moreover, NLRP3 was indispensable for PA-induced inflammasome activation. In contrast, TXNIP exerted an anti-inflammatory effect in this process by suppressing the expression of NLRP3. Our observations in NASH mice models and PA-stimulated KCs were consistent with the NLRP3 inflammasome activation observed in human samples.

## DISCUSSION

NASH is an increasing cause of liver disease necessitating liver transplantation and predicted to become the primary indication for liver transplantation by 2020 [2, 3]. The pathogenesis of NASH has been linked to multiple mechanisms, including dietary and environmental factors, and genetics which can affect innate immunity, inflammation, hepatocellular injury and cell death [1]. Maintaining a balance between the activation and inhibition of inflammatory pathways is extremely important to allow the immune system to remove any sources of danger, without causing detrimental harm to the host [8]. The NLRP3 inflammasome and IL-1β signalling pathways in KCs have been shown to play essential roles in NASH pathogenesis [8].

In the present study, we found that FFA, as a mechanistic activator of NLRP3 inflammasome, activated NLRP3 inflammasome and induced IL-1β secretion in KCs and that the expression of NLRP3 increased in liver tissues and was accompanied by elevated levels of FFA and IL-1β in serum of NASH patients and MCD induced mice model. Furthermore, we explored the important mechanisms by which TXNIP and NLRP3 inflammasome regulate inflammatory responses during NAFLD progression, and we found that the NLRP3 inflammasome and IL-1β secretion pathway in KCs were mainly activated during the progression from NAFL to NASH in WT mice, rather than from normal liver to NAFL. However, TXNIP deficiency can exacerbate mice NAFL induced by HFD alone and lead to NASH formation, which was probably caused by losing the TXNIP suppression effect on NLRP3 and then NLRP3 inflammasome activation in the KCs from TXNIP^−/−^ mice. Consequently, we deemed that TXNIP in KCs played a protective and anti-inflammatory role by binding to and suppressing the expression of NLRP3. The *in vitro* results indicated that NLRP3 and TXNIP had opposite functions during NLRP3 inflammasome activation and IL-1β secretion in KCs induced by PA, which was in accordance with the *in vivo* findings. Previous studies have suggested a central role of mitochondria in NLRP3 inflammasome activation [22]. In addition to providing an ideal platform for the assembly of NLRP3 inflammasome, mitochondria release danger molecules such as mitochondrial DNA (mtDNA) and cardiolipin, which are able to bind and activate the NLRP3 inflammasome through ROS-dependent and ROS-independent pathways, respectively [23–25]. In this study, we also demonstrated that TXNIP and components of the NLRP3 inflammasome are mainly bound to mitochondria in KCs after PA treatment, thus suggesting that the NLRP3 inflammasome probably associate with an essential molecule released from mitochondria in response to NLRP3 activators.

The role and function of the NLRP3 inflammasome in the MCD diet-induced NASH model are controversial. Consistent with our results, it has been reported that activation of the NLRP3 inflammasome in hepatocytes and liver mononuclear cells promotes the progression of NASH [11]. However, Henao-Mejia et al. have reported that mice knockout in NLRP3, ASC, or Caspase-1 developed more severe steatosis and inflammation when they are fed with MCD diet [21]. The authors concluded that genetic inflammasome deficiency-associated dysbiosis resulted in abnormal accumulation of bacterial products in the portal circulation. The liver, which is the “first pass” organ and thus exposed to the highest concentration of portal system products such as pathogen-associated molecular patterns (PAMPs), is expected to be most vulnerable to the effects of these molecules [21]. A tissue-specific knockout model may be required to specify the role of NLRP3 inflammasome activation in future studies.

A large number of DAMPs and PAMPs can activate NLRP3 inflammasome. Given the structural diversity of these agonists, it is difficult to find that NLRP3 can directly recognize and associate with all of these DAMPs and PAMPs [26]. Therefore, the mechanisms by which these distinct agonists activate NLRP3 inflammasome are currently unclear and have recently been debated, although it is generally accepted that there are likely to be several cofactors involved in NLRP3 inflammasome activation [22, 27]. The role of TXNIP plays in NAFLD development and NLRP3 activation is unclear and has not been widely explored. It has been reported that following an increase in ROS induced by NLRP3 activators, TXNIP dissociates from TRX and binds to NLRP3, leading to NLRP3 activation, and TXNIP knockout impairs NLRP3 inflammasome activation and IL-1β secretion in bone marrow macrophages after stimulation with NLRP3 agonists [18]. Zhang et al. reported that TXNIP silencing blocked NLRP3 inflammasome activation and lipid metabolism perturbations in fructose-exposed hepatocytes, whereas antioxidants addition abrogated TXNIP induction and diminished the detrimental effects in fructose-exposed hepatocytes and rat livers [28]. However, our results both *in vivo* and *in vitro* showed that TXNIP deficiency exacerbated NAFL and resulted in NASH formation, and that NLRP3 inflammasome activation and IL-1β secretion in KCs were not blocked but were enhanced in the absence of TXNIP, thus suggesting that TXNIP suppresses the expression and function of NLRP3 inflammasome by binding to NLRP3.

In summary, the present study demonstrates that TXNIP, NLRP3 inflammasome activation, and subsequent IL-1β secretion in KCs are involved in NAFLD development. TXNIP and NLRP3 play opposite roles during different stages of NAFLD development. TXNIP plays a protective and anti-inflammatory role by binding and suppressing NLRP3 mainly during the formation of NAFL, whereas NLRP3 plays a pro-inflammatory role in the progression of NASH. Our findings reveal novel links between TXNIP and NLRP3 and suggest potential therapeutic targets for NAFLD progression.

## MATERIALS AND METHODS

### Human samples

This study was conducted in accordance with the ethical guidelines of the 1975 Declaration of Helsinki and was approved by the Committee for Human Subjects of Chongqing Medical University. Informed consent was obtained from all subjects. Human liver samples (*n* = 17) were obtained from patients with suspected NAFLD. The NAFLD diagnoses were provided by two pathologists according to the NAFLD activity score (NAS) system [29].

### Animals and diets

TXNIP^−/−^ and NLRP3^−/−^ male mice aged 8 weeks purchased from Jackson Laboratories (Bar Harbor, ME, USA) were bred in the laboratory animal research centre of Chongqing Medical University (Chongqing, China). WT C57BL/6 mice were obtained from the laboratory animal research centre of Chongqing Medical University. WT, TXNIP^−/−^ and NLRP3^−/−^ mice were divided into three dietary groups: normal diet (ND) or HFD (D12492, Research Diets, USA) for 8 weeks or MCD diet (A02082002B, Research Diets, USA) for 4 weeks. Each of these nine groups contained 6 mice. The KCs of the above groups were isolated and directly analysed. For *in vitro* studies, we purified and cultured primary KCs from a second batch of WT, TXNIP^−/−^ and NLRP3^−/−^ mice fed with ND (*n* = 6 animals per experimental group). All animals were housed under specific-pathogen-free conditions and received humane care in compliance with the guidelines of the institution as outlined in the guide for the care and use of laboratory animals prepared by the National Academy of Sciences, and the methods were carried out in accordance with the relevant guidelines. The experimental protocols were approved by the committee for animal experimentation at Chongqing Medical University.

### Histological analysis

Sections of formalin-fixed livers were stained with hematoxylin-eosin (HE), Oil Red O, Sirius Red, and immunohistochemical staining for F4/80 (ab16911, Abcam, UK), NLRP3 (sc-66846, Santa Cruz, USA) and TXNIP (sc-33099, Santa Cruz, USA). The quantitative immunohistochemical staining values (QISVs) were calculated as the integrated OD divided by the total area occupied by the brown cells in each slide. The ultrastructures of the liver tissue were observed using a transmission electron microscopy (TEM, Hitachi, Japan).

### Serum analysis

Serum aminotransferase levels were determined according to the rate method. Serum levels of FFA were measured using an enzymatic method (Clinimate NEFA kit, Sekisui Medical Company, Japan). Serum IL-1β levels were determined by chemiluminescence (BioRad, USA). Serum lipid level was measured with enzymatic color tests (serum triglyceride determination kit, Sigma-Aldrich, UK).

### *In vitro* experiments

Primary KCs isolated from WT, TXNIP^−/−^ and NLRP3^−/−^ mice livers were cultured as previously described [30]. Cell viability (> 90%) was evaluated by trypan blue exclusion and KC phagocytic activity was determined by ink swallowing experiment. KCs were randomly divided into two groups: control group (CON group) and PA group (palmitic acid, 0.32 mM). The cells were starved for 12 h and stimulated for an additional 12 h. Then, inflammasome proteins and their interactions were further analysed.

### Mitochondria/cytosol protein fractionation analysis

The mitochondrial and cytosolic KC fractions were extracted using a ProteoExtract^®^ Cytosol/Mitochondria Fractionation Kit (QIA88, Millipore, Calbiochem, USA) as per manufacturers’ instructions. Cytochrome C oxidase subunit 4 (COX4) was used as the control for the mitochondrial fraction. Equivalent protein amounts from the cytosolic and mitochondrial fractions were analysed by western blotting.

### Western blotting

Total protein was extracted in cell lysis buffer (R0278, RIPA buffer, Sigma, UK) and the protein concentration was determined. The protein concentration was determined with a BCA Protein Assay Kit (23227, Thermo, UK). Equal amounts of proteins per sample were separated by electrophoresis and transferred onto polyvinylidene fluoride membranes. The membranes were then blocked for 1 h with 5% albumin bovine and incubated with primary antibodies specific for TXNIP (sc-33099, Santa Cruz, USA), NLRP3 (sc-66846, Santa Cruz, USA), ASC (sc-22514-R, Santa Cruz, USA) and Caspase-1 (sc-56036, Santa Cruz, USA) at 4°C overnight. The membranes were washed and incubated with the secondary antibodies. Protein expression was assessed by chemiluminescence. Relative amounts of protein were quantified based on the relative densities of the protein bands, by using an image analysis system (Bio-Rad Gel Doc 2000, USA).

### Co-immunoprecipitation

The interactions between TXNIP and NLRP3 inflammasome proteins in KCs were analysed by co-immunoprecipitation (Co-IP). Briefly, total protein was extracted in cell IP lysis buffer (87787, Thermo Scientific Pierce, USA). The protein concentration was determined with a BCA Protein Assay Kit (23227, Thermo, UK), and consistent protein amounts were used for each group. Then, each sample was equally divided into IP and IgG groups, and 500 μg of protein in each of the IP and IgG groups was incubated with 5 μg of NLRP3 or 5 μg IgG antibody (AP162, Millipore, USA) at 4°C overnight with continuous mixing. Fifty microliters of PureProteome Protein A Magnetic Beads (LSKMAGA10, Millipore, USA) was washed with PBST and added to the lysed pre-formed antibody-antigen complex. The mixture was incubated for 2 h at room temperature with continuous mixing to capture the immune complex, and the immune complex was then separated from the magnetic beads by heating at 95°C for 10 min. Finally, the interactions between TXNIP and NLRP3 in the inflammasome were detected by western blot analysis as described above.

### Nano-LC-MS/MS analysis and protein identification

High performance liquid chromatography-mass spectrometry (LC-MS) was used to confirm the Co-IP results and to identify other potential molecules. Protein bands of interest were excised from the Coomassie blue-stained gel. Each gel slice was diced into small particles (1mm*1mm*1mm) and placed into a 96-well plate. Sample preparation was followed the standard protocol as described previously [31]. Gel slices were de-stained and digested with sequencing grade trypsin (Promega, USA) at 37°C overnight. The protein digests were extracted, lyophilized and re-dissolved for nano-LC-MS/MS analysis, which were performed using the Q-Exactive (Thermo Scientific). The Maxquant Software was applied for protein identification and quantitation.

### Caspase-1 activity assay and cytotoxicity assays

Caspase-1 activity in KCs was determined with a colorimetric assay (R&D Systems, USA). Cell death was determined by the lactate dehydrogenase release assay using CytoTox 96 Non-Radioactive Cytotoxicity Assay kit (Promega) according to the manufacturer's instructions.

### Immunofluorescence analysis

The protein expression levels of NLRP3, ASC and Caspase-1 in KCs were detected by immunofluorescence (IF). IF was performed according to the manufacturer's instructions. In detail, the cells were fixed with 4% paraformaldehyde for 15 min and then permeated in 0.2% Triton X-100 in PBS for 10 min. The cells were then blocked with 10% serum in PBS for 1 h and incubated with the primary antibody described above (1:200) overnight at 4°C. The cells were then incubated with the secondary antibody (1:200) (Alexa Fluor^®^ 488 conjugate, Alexa Fluor^®^ 555 conjugate, Alexa Fluor^®^ 647 conjugate secondary antibody, Invitrogen, UK) for 1 h. After been washed with PBS, the cells were covered with the mounting medium, and the slides were viewed by a confocal microscopy (Nikon, A1+, Japan), ensuring non-saturating identical equipment settings for intensity comparisons among different groups. The Mander's overlap coefficient values were analysed for the degree of co-localization.

### Statistical analysis

The values are expressed as the mean ± SD (standard deviation) and compared by one-way ANOVA followed by Tukey post-hoc test when significant for the comparisons of interest using the SPSS statistical package, version 18.0 (International Business Machines, Armonk, NY, USA). *P* < 0.05 was considered to be significant.

## SUPPLEMENTARY MATERIALS FIGURES



## Abbreviations

NAFLD: Non-alcoholic fatty liver disease
NLRP3: NACHT, LRR, and PYD domains-containing protein 3
NAFL: Non-alcoholic fatty liver
NASH: non-alcoholic steatohepatitis
FFA: free fatty acid
LPS: lipopolysaccharide
PA: palmitic acid
KCs: Kupffer cells
HFD: high-fat diet
MCD: methionine choline deficient
WT: wild type
TXNIP: thioredoxin-interacting protein
TEM: transmission electron microscope
Co-IP: co-immunoprecipitation
LC-MS: liquid chromatography-mass spectrometry
DAMPs: danger-associated molecular patterns
PAMPs: pathogen-associated molecular patterns.
