# Supplementary material for: Inhibition of NLRP3 inflammasome by thioredoxin-interacting protein in mouse Kupffer cells as a regulatory mechanism for non-alcoholic fatty liver disease development
Source: Oncotarget. 2017 Apr 27;8(23):37657–72. doi: 10.18632/oncotarget.17489 (PMC5514938; doi:10.18632/oncotarget.17489)
Supplement: Supplementary file 1 [file oncotarget-08-37657-s001.pdf]

# Inhibition of NLRP3 inflammasome by thioredoxin-interacting protein in mouse Kupffer cells as a regulatory mechanism for non-alcoholic fatty liver disease development

## Supplementary Materials

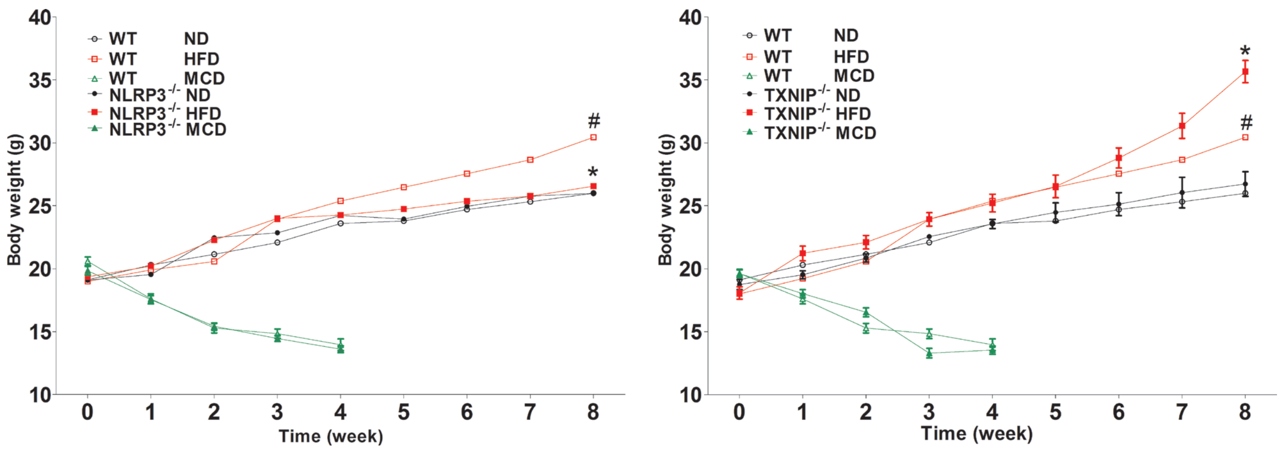


**Supplementary Figure 1: Animal body weights of WT, NLRP3$^{-/-}$ and TXNIP$^{-/-}$ mice during the interventions.**

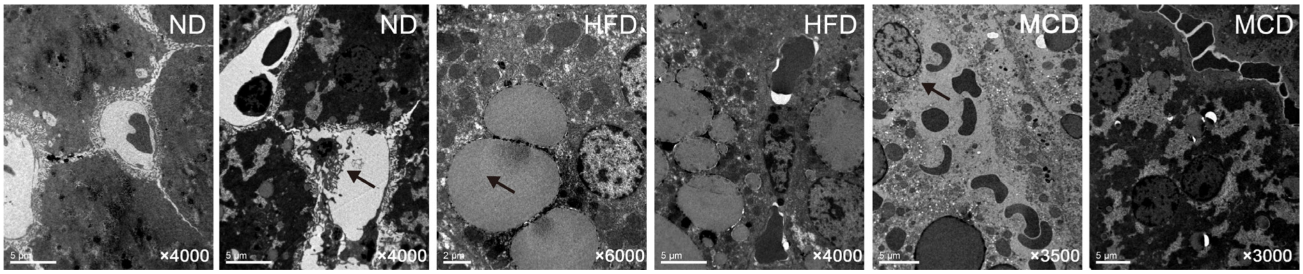


**Supplementary Figure 2: TEM images of WT mouse livers in the ND, HFD and MCD groups.** Arrows: KCs, lipid droplets, damaged hepatocytes. The original magnification and scale bars are labelled in each picture.
